# Looking into the future ten years later: big full containerships and their arrival to south American ports

**DOI:** 10.1186/s41072-021-00083-5

**Published:** 2021-03-06

**Authors:** Ricardo J. Sánchez, Daniel E. Perrotti, Alejandra Gomez Paz Fort

**Affiliations:** 1 UN-ECLAC, Santiago, Chile; 2 Pontificia Universidad Católica Argentina, Buenos Aires, Argentina; 3 UN-ECLAC, Washington, DC, USA; 4 Universidad de Buenos Aires Argentina, Buenos Aires, Argentina

**Keywords:** Shipping, ULCS, Port, Forecast, Decoupling, Planning

## Abstract

Since 2006, when the Emma Maersk broke into the world of shipping, the growth in containership size has remained a continuous trend.For the last 14 years, since 2006, the enlargement of fullcontainerships size has remained a continuous trend since Emma Maersk broke into the world of shipping. This process - that also affected north-south trades - has crucial implications in the shipping business, particularly in the planning of ports and its services and related activities. This paper analyses the global increase in vessel size and forecasts larger vessels’ arrival to South American coasts. The paper analyses evidence since 2006 to understand the factors behind the trend for bigger ships (fleets between 18,000 and 24,000 TEU) and introduce a validated methodology for the prediction of the size of container ships. Experts presented a consensus vision in which factors associated with infrastructure, economics, technology, and the environment play a crucial role in driving the trend. Next, the paper presents a methodology for forecasting the size of containerships and applies it to Latin America’s trade. The models include two alternative thresholds for the dependent variables (1310 ft LOA and 18,000 TEU of nominal capacity) that are controlled by cascading effect (i.e., the size gap between Latin America and the world’s main trade routes), and the economic activity at the destination countries (represented by port activity). Finally, the conclusions highlight the forecast’s call to take action on infrastructure planning and investments, analyzing issues such as “economies of scale,” concentration, or entry barriers. Overall, the paper warns about the importance of efficient medium-term planning in the port industry to maximize its economic impact.

## Introduction

The growth in container ships’ size has been continuous, particularly since 2006, when the Emma Maersk broke into the world of sea shipping. Drivers of large container vessels’ growth in the main trades will be discussed later, jointly with “cascading” due to that and an essential determinant of large container vessels’ arrival to Latin America and other secondary and tertiary trade destinations.

The present study seeks to analyze the growth of vessel size globally, the factors that condition it, and the timespan before bigger ships arrive at the South American shores. Historically, knowledge of factors affecting growth trends in large containerships across main trade routes has proven directly applicable in forecasting the size of future container ships of Latin America (Sánchez and Perrotti 2012). Cascading effects one of the main factors considered in the model is forecasting the size of container ships in Latin America. To such ends, the second section reviews, at a global scale, the main trends in the international sea container shipping industry, among which the almost continuous growth in the size of vessels is one of the main. The same section analyzes the evidence from 2006 up to the present day and the biggest fleet (18,000 to 24,000 TEU) to better understand the factors that drove the increasing vessel size trend.

The third section offers a consensus vision by experts that accounts for the factors related to infrastructure, economy, technology, and the environment, which impact the trend towards larger ships’ growth. Moreover, the fourth section revises the importance of having scientifically validated forecasts for properly undertaking the planning of infrastructures and proposes a methodology for predicting container ships’ maximum size on a global scale.

The following instance of this survey (section five) involves undertaking a predictive study on the arrival of larger ships to Latin America. Two alternatives for the dependent variable are used (1310 ft LOA and 18.000 TEU of nominal capacity) controlled by cascading, the gap in maximum sizes between Latin America and the leading global trade routes. The econometric analysis is an update of the one undertaken by Sánchez and Perrotti 2012, which succeeded in anticipating by 7 years the arrival of 13.500 TEU ships at South America.

Finally, some final thoughts are provided, highlighting the authors’ predictive studies’ soundness and the consequences of infrastructure planning and investments. Among the causes and effects, issues such as “economies of scale,” concentration, or entry barriers are assessed. The container vessel size growth has a strategic implication on the shipping business and the planning of ports and associated activities. Consequently, the results are interesting for understanding the need for efficient medium-term planning of the ports industry and logistics, maximizing the regional economy’s benefits.

## The ultra large (fully cellular) containerships are part of the main trends of the container shipping industry

**Fig. 1:**
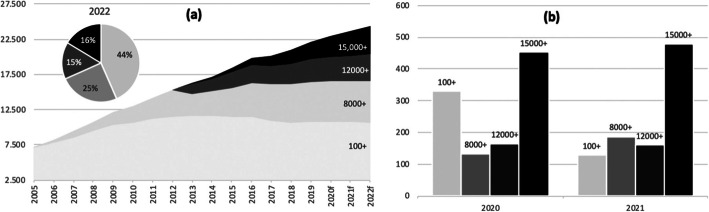
**a** Gigantism, evolution 2005–2022(f) and **b** New coming deliveries. Source: authors, based on Dynaliners, several issues. Notes: both vertical axes refer to the nominal capacity of the world fleet measured in 000 TEU

Since 2006, consistent growth has become an established trend in the shipping industry. In 1988 the first post Panamax appeared “President Truman” (275 m in length, 39 m in breadth, and 12,5 m in draft), in 2006 a significant ship as “Largest-ever container ship” appeared, Emma Maersk (397 m in length, 56 m in breadth, and 15 m in draft). The following figure displays the evolution of gigantism in ships that compose the containerized fleet between 2006 and 2022 (f); clearly, the ships of up to 11,999 TEU show stagnation or decline while those of over 12,000 or 15,000 TEU come to dominate the scene. Figure 1b allows appreciating larger ships’ pre-eminence through ships’ construction orders to be delivered during 2020 and 2021.

The data which informs Fig. 1b indicates that 25% of the construction orders, in the number of ships, correspond to ships of over 12,000 TEU (composed by 15% of the ordered fleet which is vessels of over 15,000 TEU, and 10% for those in the 12,000–14,999 range). Such 25% of units compose 67% of the global ordered fleet in terms of nominal capacity. The sum of all ships over 12.000 TEU amounts for 67% of the total ordered fleet (47%, 15,000+ and 20%, 12,000-14,999). Under such conditions, by the beginning of 2022, 16% of the whole fleet will be 15,000+ and 15% of the 12,000+.

**Fig. 2:**
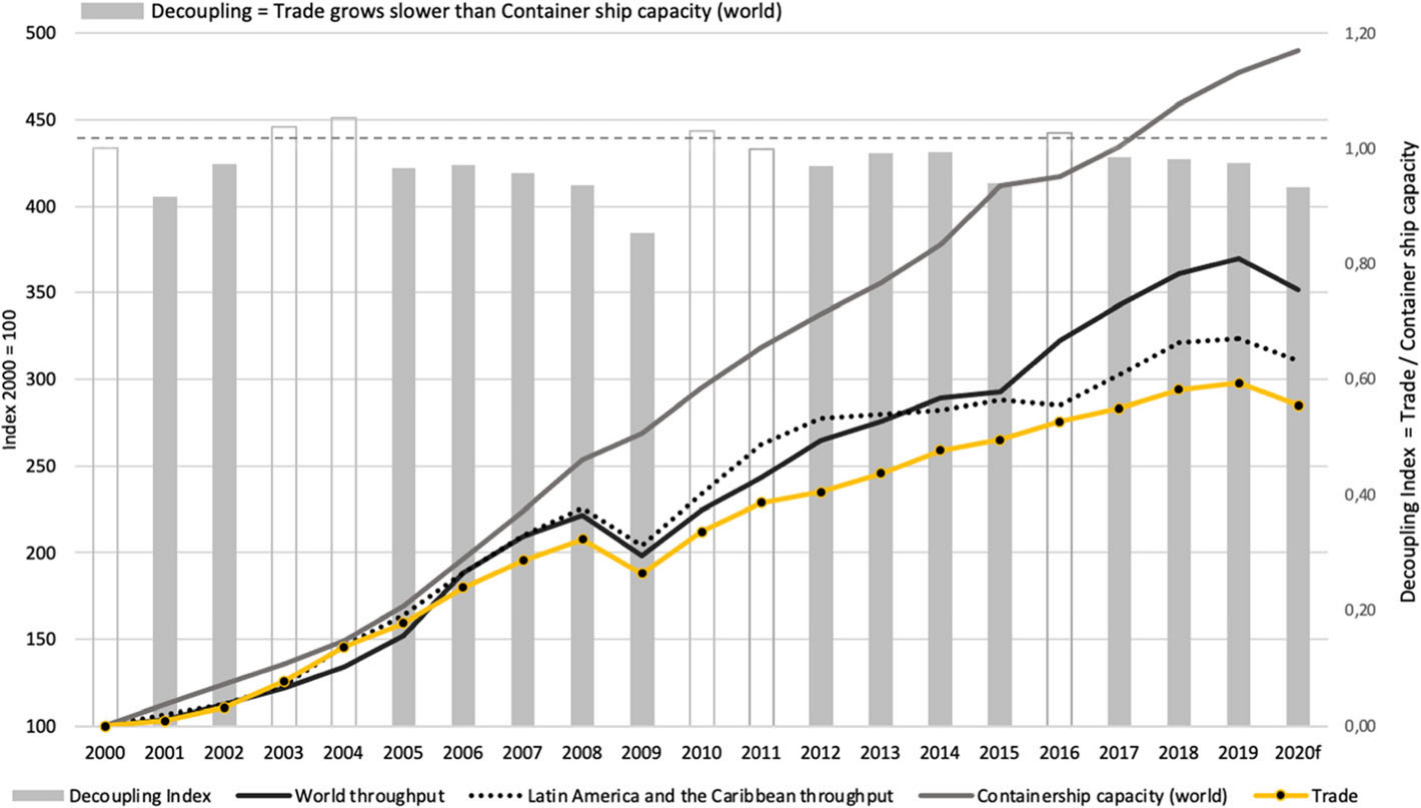
Decoupling: trade, throughput, and nominal capacity of the fleet. Source: updated from Barleta and Sanchez (2018). Note: 2020f according to Clarkson March 2020

**Fig. 3:**
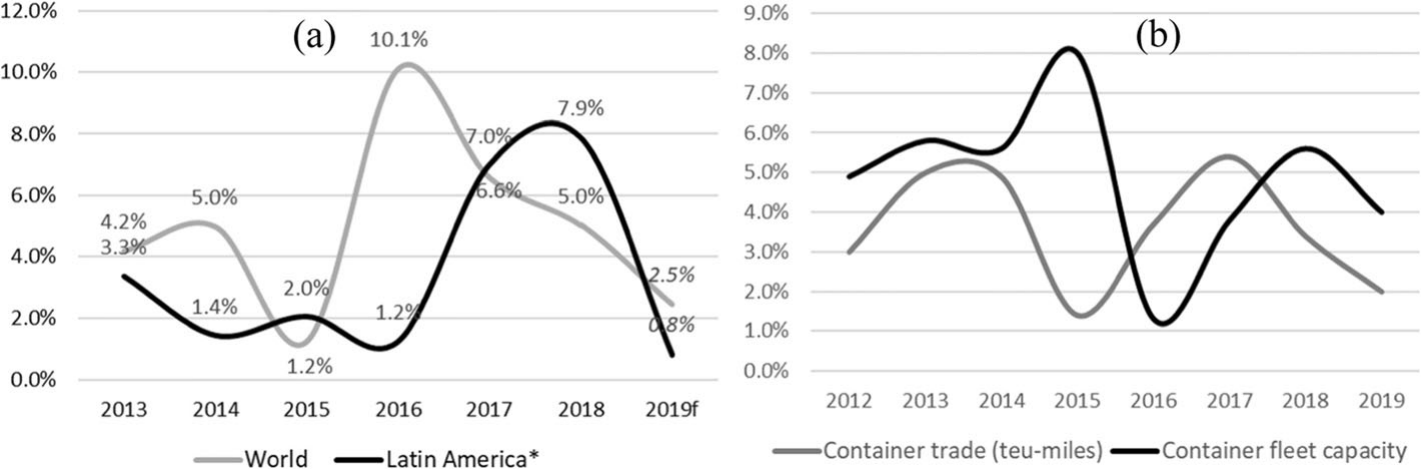
Trade traffic volatility and overcapacity. Source: authors’ elaboration

The following charts showcase other tendencies in the global industry related to ships’ gigantisms, such as decoupling, which stems from the combination of the volatility of sea trade with the overcapacity in the shipping industry presented in Figs. 2, 3a, and b. As regards the first of the mentioned phenomena, Fig. 2 allows seeing that the evolution of the fleet’s growth (by capacity) was like that of the global and Latin American throughput. Such a trend was broken in 2009 with a great crisis. Since that time, the gap is growing between expanding the fleet’s nominal capacity and the actual movement at ports. In other words, the supply is increasing more than the demand is. Figure 2 showcases a “decoupling index,” a ratio of the change in trade rate to the change in the rate of Container ship capacity over a while. Decoupling index in year n = chain index of trade-in year n / chain index of Container ship capacity in year n. A Decoupling index of < 1 suggests that trade grows at a slower rate than container ship capacity. The decoupling index’s methodology is based on Wang (2011), who constructed decoupling indicators to analyze the changing relationship between economic development and energy consumption. Between 2000 to 2010, the decoupling index’s overall trend is < 1—this evidence decouples the maritime business.

Decoupling results from the combination of the fleet’s overexpansion (Fig. 3b) and the significant volatility of sea trade (Fig. 3a). In Fig. 3b, adding the areas between the capacity and trade curves (measured in TEU-miles) shows the surveyed period’s accumulated overcapacity. The combination of both in decoupling is verified that between 2010 and 2019, the fleet measured in nominal capacity grew at a 5.5% CAGR, but maritime trade did so at 3.8%. Regarding the evolution from 2006 to 2019, those figures were at 7.1% and 5.8%, respectively.

**Fig. 4:**
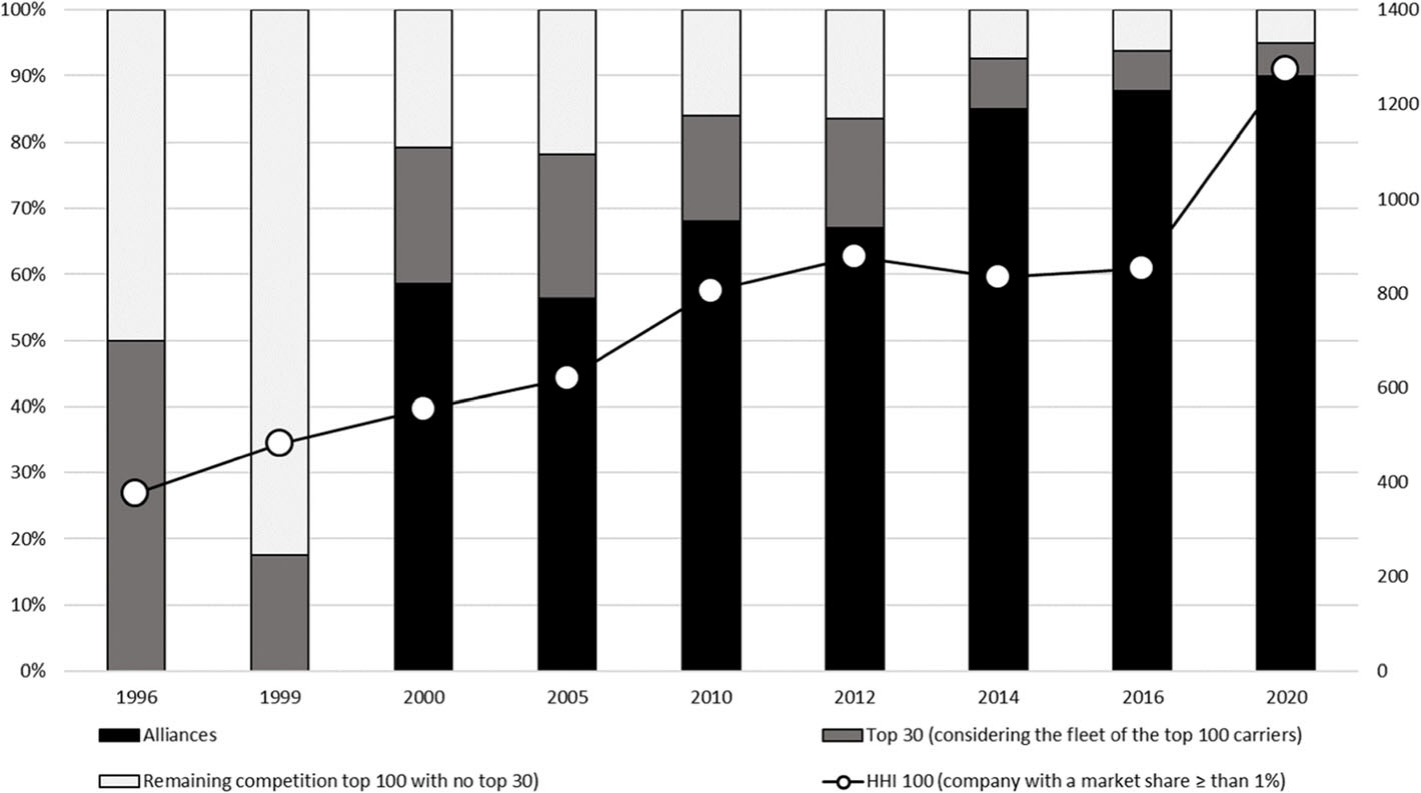
Concentration in the shipping industry. Source: Eliana P. Barleta and Ricardo J Sanchez, UN-ECLAC

Finally, the current industry’s much relevant condition turns out to be its high degree of concentration, brought on through mergers and acquisitions and the formation of alliances, as the fourth graph shows. Many authors have stated that gigantism aids the process of industrial concentration. Concentration accelerated from 2009 and particularly in a contemporary fashion with decoupling, volatility, and overcapacity. Since 2012, there is a rapid increase of the shares held by alliances within the fleet’s total nominal capacity. In Figure 4 it can be seen that ...The 30 leading companies’ capacity not participating in an alliance decreases, as does that of the remaining 70 companies in the yearly top 100. There is also a marked increase in the Herfindahl – Hirschman Index (world level), already approaching that of the moderately concentrated industry, according to the guidelines of the United States Federal Trade Commission’s Bureau of Competition.

In summary, the increase in fully cellular containerships has been one of the shipping industry’s main characteristics since 2006. The pace of growth has also been notorious. Containerships have grown faster than could be expected a few years ago when almost no one expected for a vessel of nearly 24,000 TEU to find itself sailing at present. Different researchers, including those the authors of this essay, have sought to understand how vessels’ evolution would occur with the hope for previous planning that would allow the regions to adapt to the new trends to sustain competitiveness.

Consequently, it is essential to understand the vessels’ real causes, bigger and bigger coming to different markets worldwide. While increased vessel size allows for deriving economies of scale, vessels, markets, and corporate strategies have undergone significant changes in the recent past (Sánchez and Wilmsmeier 2017).

The evolution in ship sizes is indicating that pursuing economies of scale continue to form an important goal of broader corporate strategies in shipping jointly with M&A and commercial and operative agreements: “The economic rationality for mergers and acquisitions is rooted in the objective to size, growth, economies of scale, market share and market power. Co-operation between carriers serves to secure economies of scale, to achieve critical mass in the scale of operation and to spread the high level of risk associated with investments in ships” (Notteboom et al. 2009, pp. 3,4).

As it has been said before, keywords of the enlargement process are economies of scale, markets reconfiguration, and corporate strategies. However, an emerging question is whether the simple principle of economies of scale is still a valid argument, or if current tendencies need a broader and more complex discussion to understand the continued increase of vessels even in stagnating and sometimes declining markets (Sánchez and Wilmsmeier 2017). Hence, other elements are essential to be considered drivers at the same level of seeking economies of scale and the corporate strategies in shipping, which are later discussed in this paper (see Fig. 6). This includes the expansion of arteries, globalization of production and consumption, and environmental factors. In this sense, for Latin America and the Caribbean, the Panama Canal expansion started in 2006.

Simultaneously, fully cellular containerships’ continued growth has fuelled a pattern of ship redeployment, where the new biggest are directed to the main world trade routes (mainly Asia-Europe). This “cascading” process of the “old biggest” has allowed the appearance of large containerships on secondary trade routes, among which Latin America stands out, and even tertiaries.

Seeking economies of scale has led the shipping industry to create larger companies through mergers and/or strategic alliances (Sánchez and Wilmsmeier 2017; Huang and Yoshida 2013) and has consequently made elevated entry barriers: “When scale of alliance becomes bigger, the oligopolistic or monopolistic characteristics would emerge rapidly such as higher barriers of market entry, huge capital investment and pressure on freight rates because every alliance provides exact same service” (Huang and Yoshida^ 2013^, p 4). Agarwal and Ergun (2010) build on the argument of economies of scale, but also refer to the fact that “the capacity on a ship is perishable, as once the ship leaves the port the capacity becomes unusable until it reaches a loading port again” (Agarwal and Ergun 2010, p 4). Nevertheless, other authors have highlighted concerns about the rationale behind the binomial ‘large vessels-economies of scale’: “the immediate result of the mega-ship buildings is an over-tonnaging of the world’s major liner routes” (Lim 1998, p 1).

Another positively taken driver is the environmental one; in the United Nations’ 2030 Agenda for Sustainable development, the maritime business assumes the protagonist. Energy consumption per container is already problematic. New technologies appear, too, some of which are concerned with ships and port facilities. This subject is discussed in the next chapter.

In addition to the drivers identified before, it should be considered that historically exists a high degree of uncertainty, which relates to the diversity of interacting factors in the industry (such as social, political, and fundamentally economic aspects). In the present day, the high degree of interdependence originated from globalization might cause a specific event, limited to a single actor and/or market, to trigger a massive impact situation (including economic, political, and/or social crises) until the process reaches a new balance. The referred interdependence is exemplified by the current pandemic, which has a substantial impact on maritime shipping. However, coronavirus is not the first historical event to reconfigure the balance in maritime shipping. Many previous events have been exogenous to maritime shipping, which suddenly forced substantial changes in schedules, commercial practices, or even the configuration of vessels itself. The two breakages of the Suez Canal, the first in 1956 with the closure of transits, and the second in 1967, which lead to the emergence of supertankers, are two commonly known occurrences. The construction of the Panamax vessel in 1972, which was a landmark for the size of container ships; the first and second oil crises of 1973 and 1979, among other incidents, also appear on a prospective non-exhaustive list of relevant historical facts. Among other authors, Cipoletta and y Sánchez (2009) and Stopford (2009) prove the impact of economic events related to sea shipping, while Gomez Paz (2013) provides a historical series of events that affected sea shipping.

Who could anticipate the announcement of the 18.000 TEU Triple E Maersk Mc-Kinney Møller ship, which became operational in 2013? It was difficult to predict that after the financial crisis of 2008–2009, a vessel larger than the Emma Maersk of approximately 14.000 TEU, which became operational in the year 2006 and went on to be one of the largest vessels until 2013, would be launched. During those years, the prediction of larger vessels had different visions; there were influencing factors: the low tendency in trade and an increase in ship orders (UNCTAD 2009), implications at ports (Penfold 2008), and transport infrastructures such as the New Panama Canal, with a layout that would not allow at first to pass the Emma Maersk through; all these factors slowed down the tendency. However, the hub ports such as Rotterdam and Le Havre adapted their layouts to the new challenges presented by larger ships, and other factors were at hands such as the shipping liner concentration (SYS 2009), economies of scale (Dohlie 2009), decrease in ship construction prices (Barry Rogliano Sales 2010-2020), concerns and a tendency for more sustainable vessels (Dohlie 2010). These factors encouraged the trend of growth in larger container ships.

Considering these visions, Gomez Paz (2013) based on a prospective method based on a semi-quantitative *Delphi* methodology (experts consensus) and a quantitative model (dynamic model, “predictive game”), predicted future scenarios and revealed that the size of vessels was limited mainly by the depth of canals and the dredging in ports and that other factors such as the new CO2 restriction measures, the price of oil, certain economic magnitudes, costs by a unit of transport and the concentration of shipping lines, drove the trend towards larger vessels, breaking the equilibrium between supply and demand. The survey results showed that by the year 2032, ships would be sailing with a capacity of between 20,000 and 26,500 TEU (minimum and maximum scenarios, respectively).

West Coast of South America expects the arrival of 400-m-long (Portal Portuario 2020). A study by Sánchez and Perrotti (2012) already estimated the arrival of large ships in Latin America, observing that the large ships that sailed on the main routes needed for progressively fewer years to reach the coasts of South America. The survey already estimated a 13,500 TEU ship’s arrival before 2020, verified by their appearance as early as 2017.

## Factors that affect the growth trend of large fullcontainerships

The ambition of producing ever-larger ships is pervasive. Wijnolst et al. (1999) encouraged thinking about large containerships, such as the Malacca-Max, which would be limited only by the Malacca strait draft. A constant effort for maximizing ships was made, including their technological enhancement. In 2008, Korean shipyard STX announced it had designed a 22,000 TEU large containership (STX 2008). Meanwhile, in 2011 rumor spread that Maersk would order 18.000 TEU ships, and Alphaliner forecasted 20,000 TEU ships for 2014 (Alphaliner 2011).

All forecasts for larger ships have been exceeded already. Currently, ships over 24,000 TEU sail across the globe, adapted to current conditions and technology. In July 2019, Alphaliner (2019) presented data on the world’s largest container vessels: 89 vessels ranging from 18,000 to 24,000 TEU exist already, belonging to MAERSK (35%), MSC (20%), COSCO (19%), EVERGREEN (12%), MOL (7%) and UASC (7%), and underline that 32 ships “MGX-24” or Megamax-24 (ships with 24 rows and 61.5 m breadth) have been ordered by HMM, MSC, and CMA-CGM. It is worth noting that ships on service in the 18,000–24,000 TEU range are 400 m in length, 58.6–61.5 m in breadth, and 16–16.5 m in the draft. The MSC GÜLSÜN, a landmark ship, has a capacity of 23,756 TEU and includes new technology to prevent accidents, optimize space, and protect the ocean and the environment (Russo 2018).

**Fig. 5:**
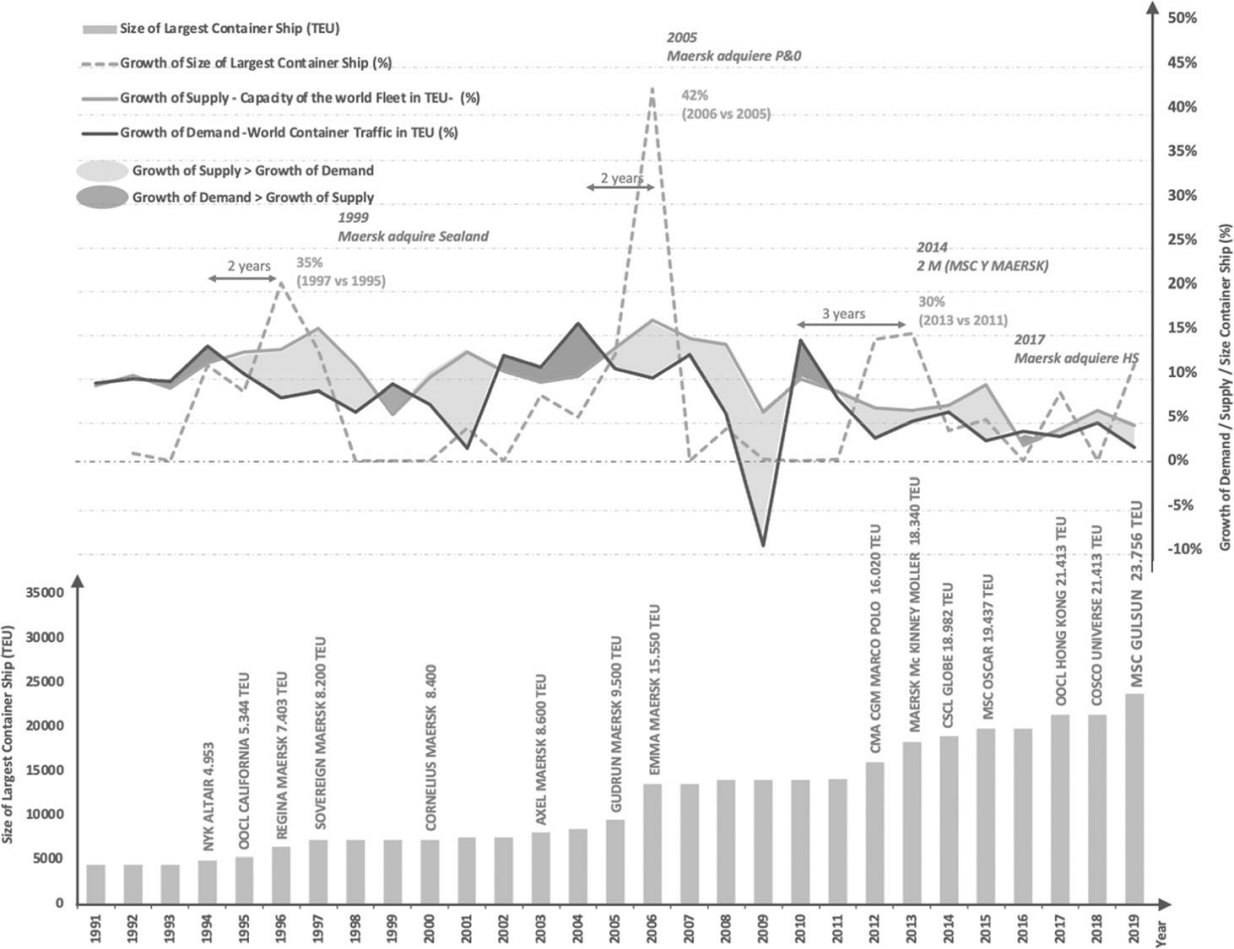
Increase in container ship size about variation in fleet and trade traffic. Source: author. Notes: 1. Remarkable Mergers & Acquisitions; 2. Total fleet, on service, and idle. 3. Ship capacity is estimated. 4. In 2012, CMA-CGM Marco Polo was delivered and on service. Source: Author, based on Gomez Paz (2013);Clarkson (1998–2020); Alphaliner (2011, 2014, 2019, 2020); Barry, Rogliano, Sales (2010-2020)

For the last 30 years (since 1990), significant ships appeared; Fig. 5 shows trends between demand -trade traffic-, supply -fleet- and the size of large containerships, highlighting the significant vessels. It is worth noting that the largest increases in ship size occurred 2 years after a period where the growth of demand, expressed as world container trade traffic in TEU, exceeded the growth of supply, measured as the world fleet’s capacity in TEU. However, this rule stopped holding past a specific point in time; thus, it is unreasonable to believe that other factors affected the overall trend. Figure 5 also showcases surplus and shortage periods that lead to overcapacity, as shown in Fig. 3b. Moreover, most relevant mergers and acquisitions coincided with a leap in the size of vessels. Figure 5 -updated to 2019- is part of Gomez Paz (2013) pre-forecast study on factors that previously affected large ships’ growth trends.

**Fig. 6:**
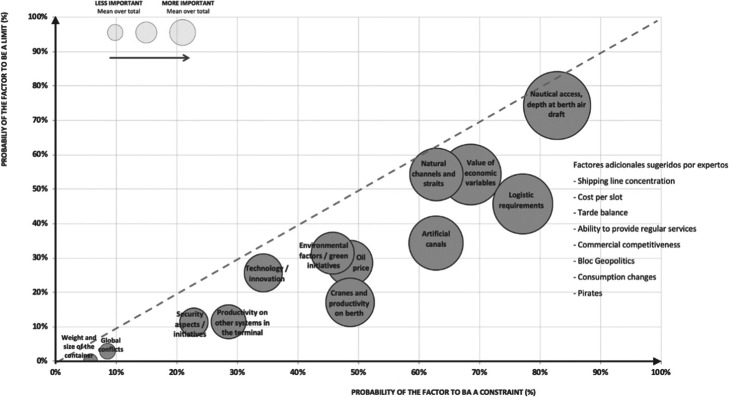
“Experts’group vision” of the factors that will constrain the growth trend of large containerships in the next 20 years, given in 2011. Source: (Gomez Paz et al. 2015). Updated with additional factors pointed out by the experts in 2011

In addition to Fig. 5, pre-forecast analysis -mind maps, timelines- helps identify and characterize several inputs for the Delphi method, allowing arriving at an expert consensus through successive surveys. This “Experts’ group vision” outlines the key factors that they agree will limit and constraint the growth trends of large containerships within the next 20 years; on surveys, the largest containership is defined as vessels over 18.000 TEU the Triple E series announced by Maersk. Gomez Paz et al. (2015) goes over the methodology and results in detail, as shown in Fig. 6.

A total of 83 experts, mainly from Europe and the Americas, most analysts and consultants were polled to arrive at an “Experts’ group vision,” shipowners, logistic operators, terminal operators, port authorities, and public institutions also participated. Three anonymous polls were carried out. At each round, experts were shown the previous round’s replies. 82% of polled individuals coincided with a joint vision by the end of the third poll. These results are Fig. 6. The 45° line helps determine what factors are likely to become limit and which to constraint, and the size of the circle the importance of each element. As the principal limit, with most probability to constraint, and more influence in the trend, is the factor associated to the nautical access, berth depth, air draft; on the other hand, with a medium likelihood to set constraint and less influence as a limit, are the issues related to the environment. Likewise, in the consecutive rounds, consultations were made on a different, previously unassessed set of factors and raised new ones such as the shipping lines concentration, consumption changes, bloc geopolitics, among others, shown on the right side of the graphic. Because they were not considered at the onset of the study, their probability is not shown in Fig. 6.

In 2020, the vision remained, reinforcing the environmental factor, which coincides with the United Nations’ 2030 Agenda for Sustainable development and its SDG 13: “Take urgent action to combat climate change and its impacts.” Pursing these goals, the International Maritime Organization -IMO- adopted an initial strategy aimed at reducing vessels’ greenhouse gas (GHG) emissions by at least 50% by 2050 (compared to 2008) while pursuing efforts towards phasing them out (IMO 2018). Facing these regulatory changes and considering that LNG is an acceptable solution, Dalian Shipbuilding Industry Company and DNV-GL signed agreements to develop a 23,000 TEU LNG-fuelled ultra-large container vessel (DNV 2018). CMA-CGM also announced the CMA CGM Jacques Saade, a 23,000 TEU sister ship of nine other LNG-fuelled ships, to be delivered in 2020 (CMA-CGM 2019).

“The main driver for ordering larger vessels is to reduce the amount of energy needed for the transport of each container, more energy efficiency lower costs as well and helps to minimize CO2 emissions, which improves profitability and reduces the environmental impact of global supply chains”, according to Russo (2018). “Can ships go larger still,” Russo (2018) quotes a reply from MSC’s new buildings project manager: “Technically speaking, there are no fundamental physical constraints, and from an operational point of view, certainly a commercial case could be made. The barrier, however, is shore-side infrastructure. We are approaching the maximum size that ports can handle.”

Stopford (2019) highlights three factors urging the maritime industry into a new era: (i) digital technology, (ii) regional realignment, and (iii) environmental emissions. For each constraint, he suggests strategies of adaptation. Penfold (2017) further focuses on port requirements: environmental, technical solutions for large vessels’ attendance, automatization, and economic and political issues. As an aside, it is worth noting that ports are being adapted to new trends. For example, UNCTAD (2009) summarizes a selection of investment projects between 2017 and 2019 and stresses that their objectives align to develop and improve ports’ infrastructure and services. The TUAS Next Generation Port project, undertaken by the Port of Singapore Authority, is noteworthy. The project aims at an intelligent, safe, green, and community-oriented port that should accommodate mega container ships in the long term, thanks to a depth of 23 m (MPA Singapore 2020). Sánchez and Barleta (2018) studied the trend of containerization. They noticed risks for the ports, facing the prospects of a slow increase in the container throughputs and global trends showing an environment of rivalry and concentration.

Other points of view have been addressed. Haralambides (2019) points out the economy of scale in ships and the diseconomy of scale across ports. Wei and Hui (2019) brings up the issue that sea traffic has to be considered besides inland traffic. Recently Ge et al. (2019) evaluated the economic, operational, and environmental conditions and expectations shipping companies are likely to push the ultra-large containership (ULCS) from 18,000–20,000 TEU to 25,000 TEU and concluded that economies of scale are achieved at 25,000 TEU, barring events of low freight rates and low load factors.

Cargo demand between shipping endpoints impacts maritime transport. In the outcome of the mentioned research, Ge et al. (2019), the load factor is affected by fleet capacity and traffic volume. Thus, decreases in traffic volume impact load factors and is likely to lead to scenarios different from those forecasted. Not long ago, after the 2008 crisis, we recall pictures with many idle ships and, at the same time, slow steaming adopted by the shipping lines.

Economies of scale bring other issues into consideration. Sanchez and Wilmsmeier et al. (2017) amend traditional analyses of maritime transport to incorporate economies of scale, economies of scope and density economies concepts, relevant to the present feature of the maritime transport, whereas the transport services offered by the shipping lines go from different types of boxes, with varying rates of the tariff, to different geographical scope, with larger units and an overcapacity, agreements between shipping lines VSA, to achieve economies in the services, discussions with the port terminals, among other factors, turn into myths, long-held beliefs of the maritime industry. All these statements create new challenges for the ports: although we notice a growth in ship’s sizes, this growth does not come in with an increase in throughput, involving port infrastructure and superstructure investments; on the other hand, opportunities for new businesses are created, which require capital to be able to offer new services. New challenges for the ports that they have to lose customers lead to more significant financial and operational risks in port planning.

Wilmsmeier et al. (2017) propose that economies of scale, concentration, and cyclical effects (supply/demand interaction) are all part of a “before hangover” cocktail; and explores upcoming challenges and posits that monitoring alliance behavior may be a possible cure. The hangover will spread geographically at random, with a global concentration that will impact the secondary and tertiary routes. The paper further addresses the relevance of drawing up international policies and regulations, integrated with national and regional, and port and infrastructure planning, looking into logistics chains, while considering production, financing, and marketing strategies.

Alliances between the shipping lines play an important role within the dynamic of supply and demand in maritime transport. Per Fig. 5, these relationships may hint at a correlation between the existence of alliances and the appearance of large fully cellular containerships, a relation due to the need for higher load factors to be economically viable. Vertical integration is another topic worth devoting attention to, as shipping lines are now offering logistics services beyond terminal services. These integrations are put in doubts by Brooks et al. (2019) and Haralambides (2019), both of whom submit points of view in this regard; Sánchez and Chauvet (2020) extend this topic even further to encompass subjects such as governance, concession contracts about infrastructure, private-public associations, and its main characteristics, and defense of competition, where they highlight the importance of the institutions and antitrust procedures. In their report, they warn about the risks of vertical integration. Indeed, there are incomplete concession contracts that do not consider the present situation, and institutions do not seem qualified to solve these conflicts. This shortcoming could lead to unfair behaviors and results that would affect the sector’s sustainable development and the economy. Therefore, governance has to be set involving all players, not only those in the private and public sectors but also the civil society and the actors involved in the logistic chain.

## Forecasting: the importance of knowing when the next large container vessel will arrive

Quantitative forecasting is a challenging task. As was noted in the previous section, several researchers have coped with this issue; nevertheless, this task is necessary to conduct competitive plans and strategies to develop transport infrastructures. Facing uncertainty, it is encouraged to build up flexible plans for infrastructure development. UNCTAD (1985) noted the importance of flexible plans. “A key principle in planning seaport facilities, therefore, is that development plans should be as flexible as possible to allow a prompt response to changing demand.” Taneja et al. (2010) recommend Adaptive Port Planning (APP), aiming to close the gaps in traditional planning and thus achieving “flexible port infrastructures.” Van Dorsser et al. (2018) submit a new perspective, presenting an approach for developing a shared vision on Rotterdam’s port’s future development. The simulation also succeeds in planning and updating port terminals and waterways, optimizing and minimizing infrastructure investments, and keeping efficiency and security. Gomez Garay (2014) presents the tools, together with a set of satisfactory cases for infrastructure works.

Different forecast models capture future trends; Gomez Paz (2013) combines the pre-forecast analysis with the “experts group vision” to identify the factors that will satisfy constraints in the years to come, the growth trend of the ships, and a quantitative model, that gives a clue to identify the factors that will encourage, slow or set a limit on the growth trend of vessels, and asses regarding the growth tender of the factors, the size of the largest ships in the coming periods up to the year 2032.

The quantitative model based on set knowledge of them: the relationship between the factors and the growth of the ship in the past, and the estimation on the growth trend of these factors in the future, based on previous estimations; from the known relation between the factors and the size of the ships, resulting in the growth trend of the ship’s size until 2032. Higher GDP results in more shipments and a broader market for large container ships; increased port depth with improved operations means that large containers can enter the port and be more productive. Deeper channels and straits may open a new route. Fewer emissions of CO2 emissions and a low unit cost encourage large ships. A greater concentration of shipping lines provides additional opportunities for large container ships. Gomez Paz’s 2013 contribution lies in positing a dynamic model “predictive game,” a model that can be updated to account for new factors affecting growth trends. These can either encourage or limit large containerships’ growth trends and find new scenarios of ship sizes and show the factor that limits the ships’ growth tender. The dynamic model “predictive game” shows a present situation that goes to a future one that can be possible and desirable; this method and the Delphi method “experts group vision” are combined and thus validated.

Growth expectations of a variable that constrains the growth of the large ships can bring forward or delay, in a timeline, the appearance of the largest ship. In 2011, the indicators related to the economic growth -traffic demand- could be the reason for a slowdown in the evolution of ships, a perception influenced by the lack of expectation in the economy’s growth. However, this perception changed in 2013, and the model showed that an increase in the economy would encourage the growth tender of the ships, causing the appearance of a giant ship in advance.

Main factors that encourage the growth trend of the ships detected by the dynamic model: concerns about the environment, a positive forecast on the economic growth, and technological improvements were leading to savings in the transport costs. Also noticed an intense concentration among the shipping lines that determine the growth trend and the adjustment to new logistical demands and the need to improve the crane productivity, which slows the growth tender. Furthermore, it points out that the investments in extensive infrastructure for adapt navigation channels and nautical access -depth at berth, channels, and strait- is a factor that limits the growth tender of the ships.

Under the influence of these factors, a ship of 26,500 TEU was expected to be delivered between 2026 and 2032; however, the forecast was anticipated, with the appearance of a 23,500 TEU ship in 2019. The factors that encourage and limit the growth trend in ships, forecasted by the “experts’ group vision” together with the “predictive game” were verified and validated the model; however, in 2009, the harshest shock to market conditions begins to take place, a growing gap between the fleet capacity and the container throughput, as shown in Fig. 2- Decoupling, and a significant consolidation that could clarify the gap between the forecast and the facts. This is an issue of great interest for future research. The growth of the fully cellular containerships will depend on the interaction of different factors and new relations among them, the dynamic forecast tools that integrate different expert visions, and based on historical and forecast, could guide about global trends, constitute an aid tool to foresee new factors and future scenarios, and dynamically anticipate them. Next section deals with the arrival of these ships to the Latin American coasts.

## When will arrive the current largest container ship generation to Latin America?

After explaining the factors behind ships’ growth, we now focus on readers’ attention on Latin America. This section presents econometric models to predict large vessels’ arrival to the region, crucial information for efficient port planning.

This objective is raised based on Sánchez and Perrotti (2012) experience who proposed a model (which is always a simplified representation of reality), which is quite simple but which had an almost perfect fit of its predictions with what occurred later) to estimate the arrival of post-Panamax vessels to South America’s coasts, using as a dependent variable the ships’ nominal capacity (measured in TEUs).

The estimates were made using the nominal capacity as a dependent variable and the length overall (LOA) in the models below. The general idea in all models is to determine the maximum size of vessels arriving in South America. The variables used in the models are presented below.

### Variables

#### TEUs (twenty-foot equivalent unit)

Max in models 1 and 2: represent ships’ capacity through the amount of TEUs they can carry. A TEU is the load capacity of a standard 20-ft (6.1 m).

#### LOA (length overall)

Max in model 3: is the vessel’s dimension taken between the two most extreme vessel points, which is used to determine the space required for docking.

#### Port activity (pa)

Represents the amount of cargo served at the ports of the east and west coasts of South America, respectively, and is measured in TEUs. As a derived demand for economic activity, port activity shows a high correlation with GDP.

#### Gap

Denotes the difference -in %- between the maximum size (in TEUs or LOA) of ships arriving in South America and those that simultaneously navigate the main trade routes. This variable captures the cascading effect performed when the new big vessels in the main trades cascade part of the fleet to secondary trades (e.g., Latin America). The cascading effect works in practice as a coin pusher machine, where the entry of a new coin (new most giant vessels in the main trades) push and spills out other coins (older ships) to the coins pick up a bucket (secondary trades).

The following table presents summary statistics of the variables:

**Table:** 

Variable	Obs	Mean	Std. Dev.	Min	Max
Max_saw	23	6061.57	3781.98	2512.00	13,102.00
Max_sae	23	6050.17	3103.14	2591.00	11,010.00
LOAmax_saw	19	297.00	55.35	210.07	367.00
LOAmax_sae	19	300.51	30.71	266.34	339.60
D (Max_saw)	22	481.36	799.97	0	3130.00
D (Max_sae)	22	382.68	521.52	0	1545.00
D (LOAmax_saw)	18	8.72	15.51	−16.28	43.21
D (LOAmax_sae)	18	3.88	8.70	−9.59	28.40
Pa_saw	23	6.38	4.18	1.64	13.74
Pa_sae	23	8.05	3.52	1.98	13.41
GAP_LOA_SAW	23	2.46	0.67	1.47	3.53
GAP_LOA_SAE	23	2.31	0.44	1.67	3.05

### Models with nominal capacity as the dependent variable

- Model 1: is a pooled model that includes dynamic through the incorporation of the lagging dependent variable.
- Model 2: is a pooled model with error correction specification.^1^

Here it is essential to highlight those pooling models that use information from time series and cross-sectional series carry certain advantages (Podesta 2000):

i. They attenuate the problem of few observations, both for the cross-section and the time series. Generally, when having a few observations, the potential total number of explanatory variables exceeds the degrees of freedom required. With pooling models, this condition is lightened due to a joint presentation of cross-section and time variables, which allows testing a higher number of predictors in the context of multivariate analysis.
ii. These models have gained popularity since they allow investigation inside the variables, surpassing the time or cross-section analysis’s simple study. Many characteristics of the cross-section series tend to stay invariant over time. Hence the regression analysis that includes space and time dimensions tends to present a higher variability of information than time or cross-section series.
iii. A third advantage is that pooling models capture not only variations from data over time or cross-sections but also both dimensional changes simultaneously. This advantage is because a pooling model uses all the available cross-section series over time.

The estimated results are (Table 1).

**Table 1:** Model 1

Dependent Variable: MAX?			
Parameter	Coefficient	Standard Deviation	t Statistic
C	− 745.17	349.46	−2.13
MAX?(−1)	0.77	0.09	8.54
PA?	267.04	80.61	3.31
GAP?(−3)	328.26	181.63	1.81
Fixed Effects (Cross)			
SAE--C	−245.84		
SAW--C	245.84		
Adjusted R2	0.97	Sample: annual data 2000–2018	
Observations after pooling data	40		

Statistics for all parameters of both equations in Model 1 show significance – at least – above 90% of the confidence interval (Table 2).

**Table 2:** Model 2

Dependent Variable: D (MAX?)			
Parameter	Coefficient	Standard Deviation	t Statistic
MAX?(−1)	−0.35	0.13	−2.68
PA?(−1)	396.64	142.91	2.78
D (MAX?(−3))	−0.68	0.28	−2.40
D (PA?(−2))	− 169.63	6.59	−25.74
D (GAP?(−1))	− 442.42	39.58	− 11.18
D (GAP?(−3))	− 414.40	22.12	− 18.73
_SAW--C	375.23	42.00	8.93
_SAE--C	−450.01	294.63	−1.53
Adjusted R2	0.40	Sample: annual data 2000–2018	
Observations after pooling data	38		

Except for the east coast parameter’s individual effects, the rest of the parameters show statistics with the significance of – at least – above 90% of the confidence interval.

The following assumptions apply to the models (Table 3).

**Table 3:** Assumptions applied to the estimations

Scenario	Port Activity West Coast	Port Activity East Coast	Gap West Coast	Gap East Coast
Historical	6%	4%	3%	5%
Positive	7%	5%	3%	5%
Negative	5%	4%	3%	5%
Negative_2	3%	3%	3%	5%

Source: authors’ elaboration

The results suggest that the arrival of vessels of 18,000 TEUs to the South American coast would occur in the following years (Tables 4 and 5).

**Table 4:** East Coast

Scenario	Model 1	Model 2
Historical	2028	2029
Positive	2027	2028
Negative	2029	2029
Negative_2	2030	2031

Source: authors’ elaboration

**Table 5:** West Coast

Scenario	Model 1	Model 2
Historical	2024	2025
Positive	2024	2024
Negative	2025	2025
Negative_2	2027	2026

Source: authors’ elaboration

### Model with a length overall

For calculating length overall (LOA) as a dependent variable, a model like Model 2 was used (i.e., a pooled model that includes the dynamic through the lagging dependent variable). Model 3 comes in the following format^2^ (Table 6).

**Table 6:** Model 3

Dependent Variable: D (LOAMAX?)			
Parameter	Coefficient	Standard Deviation	t Statistic
C	1.88	27.15	0.07
LOAMAX?(− 1)	0.80	0.10	8.32
PA?(−1)	7.51	1.69	4.45
PA?(−3)	−6.77	1.66	−4.08
GAP_LOA?(−1)	122.85	26.88	4.57
GAP_LOA?(−2)	37.62	12.95	2.91
GAP_LOA?(−3)	−89.94	19.94	−4.51
@TREND	2.48	1.15	2.16
Fixed Effects (Cross)			
SAE--C	−2.90		
SAW--C	2.90		
Adjusted R2	0.97	Sample: annual data 2000–2018	
Observations after pooling data	32		

Except for the intercept, the rest of the parameters show statistics with acceptable significance levels (above 90% of the confidence interval).

Assumptions: Below are the assumptions used for projections with the LOA model, where the gap is in terms of differences in LOA (Table 7).

**Table 7:** Assumptions applied to the model

Scenario	Port Activity West Coast	Port Activity East Coast	Gap West Coast	Gap East Coast
Historical	6%	4%	−7%	−2%
Positive	7%	5%	−7%	−2%
Negative	5%	4%	−7%	−2%
Negative_2	3%	3%	−7%	−2%

Source: authors’ elaboration

Results: ships with a 400-m length overall would reach the South American coasts, in the different scenarios for each coast, in the following years (Table 8).

**Table 8:** Results of estimations

Scenario	West Coast	East Coast
Historical	2021	2022
Positive	2021	2022
Negative	2021	2022
Negative_2	2022	2022

Source: authors’ elaboration

### Final thoughts

The trend towards gigantism in container ships remains in force, which was reflected in the emergence in 2006 of the EMMA MAERSK -15,500 TEU-, in 2013 with the TRIPLE E MAERSK series − 18,000 TEU-, and in 2019 with the GÜLSUN -23,756- TEU, which falls within the MGX-24 category. This trend has not been foreign to Latin America, which has seen, by a cascading effect, the arrival of ships that currently reach the 14,200 TEU mark with a 367 m length.

The containerized fleet and the industry’s commercial practices have been modified over time, though not only in capacity but in technology as well, seeking to meet goals of environmental sustainability and operational and commercial efficiency: alternatives for new fuels, container management, cost reduction, digitalization, tariff differentiation and collaborative practices such as alliances and VSA. The shipping lines have also expanded their business towards vertical integration, encompassing beyond the sea shipping service, the specialized port terminals, and inland distribution. However, such changes and the emergence of larger vessels at the main routes lead to a cascading of ships on secondary and tertiary routes, which causes new challenges for ports. And for the latter to adjust and retain services, they are forced to adapt by investments in equipment, infrastructure, and superstructure to meet the challenge of larger ships; and port authorities face new demands to their roles and goals.

Apart from gigantism, a new global context presents itself: new services, with a long reach and an intense concentration, which inform new challenges to ports, particularly ports which are affected by the cascading of ships from different routes, high investments on infrastructure and the development of new trades. The impact comes to the port and affects the whole logistics chain, spreading to production, impacting sustainable development.

This paper has provided different methodologies, with robust precedents, which allow for the estimation of the size of future ships within this decade, and which will be in the future the determinants of growth for larger vessels, underlining the factors that limit the trend related to the infrastructure at ports and waterways, and in others that drive the trend towards the growth of large ships associated with environmental requirements.

Following the methodology implemented in Sánchez and Perrotti (2012), an estimation has been made of the determinants for the coming of the current global large ships to South America. The cascading effect is captured in the analysis using a variable that accounts for the gap between the primary and secondary trades. The cascading effect works in practice as a coin pusher machine, where the entry of a new coin (new most giant vessels in the main trades) push and spills out other coins (older ships) to the coins pick up a bucket (secondary trades).

The paper included two alternatives for the measuring of vessel sizes: a) the nominal capacity, where the goal of 18,000 TEU capacity ships is set, with their arrival being estimated for 2027 at the east coast and 2024 at the west coast; b) the length (LOA), with the goal of 1310 ft vessels, which was forecasted to arrive in 2021 at the east coast and 2022 at the west coast.

It should be noted that the time differences between both projections over the same phenomenon are related to technical issues, due to the 1310 ft LOA ships ranging in their nominal capacity from 15,500 TEU to 23,700 TEU, for which the arrival of vessels of greater length is not in contradiction with the arrival of ships of greater TEU capacity in subsequent years. It is, nonetheless, entirely relevant from the port planning standpoint to consider the projections of the LOA model, for it determines the physical necessities of docks, facilities, and equipment. Additionally, this paper contributes to the synergy in planning between port authorities and terminal operators, since many times the criteria used are diverse and do not facilitate the use of objective instruments that are useful for both parties, especially in Latin America where concessionaires are not always responsible for investment in infrastructure.

In this paper, two different models have been developed (nominal capacity and vessels’ LOA) – showing time differences between both projections. Those differences should be and differences related to the region which will first receive the mega vessels (ECSA or WCSA). Such differences should be considered for planning, depending on the different types of ships in the region. It is essential to consider that ships of the same LOA have various nominal capacities in TEU, which vary between the fleets operated by the different shipping lines. Therefore, both projections facilitate the port authorities’ identification of the type of ships that could arrive on each date. According to the Gomez Paz (2013) and the surveys’ results, it is possible to assume that container ships’ growth trend will also go on a global scale. However, it should be noted that the current pandemic may lessen its slope during the next few years.

It was also pointed out that the larger ships’ phenomenon has led to the decoupling of fleet capacity and trade and that such events were contemporary with industry concentration. These issues raise certain questionings on the usual explanations used to justify concentration, such as searching for economies of scale (Sánchez and Wilmsmeier 2017).

Moreover, the 2020 pandemic creates varying reactions on behalf of the shipping sector. Although it would be hasty to state a conclusive opinion, the financial resolution of the present crisis may significantly alter the degree of concentration in the industry, and through that, the speed of the growth and enlargement of the size of fleets at a global scale (and their respective impact on the region).

## Data Availability

The datasets used and/or analysed during the current study are available from the corresponding author on reasonable request.

## Footnotes

1 Before proceeding with the estimation of models 2 and 3, the corresponding stationarity and cointegration tests were performed on the series.

## References

[CR1] Agarwal R, Ergun O (2010). Network design and allocation mechanisms for carrier alliances in liner shipping. Oper Res Vol.

[CR2] Alphaliner (2011) Chart of the week “Evolution of the world’s largest containerships 1985–2011” Alphaliner Weekly Newsletter, volume 2011 Issue 4 18.01.2011 to 24.01.2011

[CR3] Alphaliner (2014) Chart of the week, “The world’s largest containership: Who are the contenders?” Alphaliner Weekly Newsletter, volume 2014 Issue 52 23.12.2014 to 29.12.2014

[CR4] Alphaliner (2019) Chart of the week “The world’s largest containerships” Alphaliner Weekly Newsletter, volume 2019 Issue 28 03.07.2019 to 09.07.2019. https://www.alphaliner.com/resources/Alphaliner_Newsletter_no_28_2019.pdf

[CR5] Alphaliner (2020) Table of the week “Containership Fleet: Key Figures (January 1st, 2020 vs. 2019)” Alphaliner Weekly Newsletter, volume 2020 Issue 1 28.12.2019 to 07.01.2020

[CR6] Barry Rogliano Sales (2010-2020) Annual Review Volumes 2010 2020. https://www.brsbrokers.com/review_archives.php

[CR7] Barleta E, Sanchez RJ (2018) Reflections about the future of containers ports, Ciencia and Tecnología Review, v.21, n 32; UNIMEP, Sao Paulo, Brazil

[CR8] Brooks M, Vanelslander T, Sys C (2019) Regulation in the liner shipping industry: pathways to a balance of interests. https://www.researchgate.net/profile/Mary_Brooks/publication/333801978_Technical_Report_Regulation_in_the_liner_shipping_industry_pathways_to_a_balance_of_interests/links/5d04ec6392851c90043da44e/Technical-Report-Regulation-in-the-liner-shipping-industry-pathways-to-a-balance-of-interests.pdf

[CR9] Cipoletta T, y Sánchez R (2009) La industria del transporte y las crisis económicas. Serie 149, Recursos naturales e infraestructura, CEPAL. https://repositorio.cepal.org/bitstream/handle/11362/6352/1/lcl3206e.pdf

[CR10] Clarkson (1998–2020) Clarkson Research Studies: Container Intelligence Monthly, ISSN: 1467–0488, volumes 1998 2020

[CR11] CMA-CGM (2019). The CMA CGM JACQUES SAADE, the world's first 23,000 TEU powered by LNG.

[CR12] DNV-GL (2018). DNV GL and DSIC sign JDP to develop LNG fuelled 23,000 TEU ultra-large container vessel.

[CR13] Dohlie K (2009) The future of the ultra-large container ship. DNV Container Ship Update N°. 3, 2009, p. 22 a 24

[CR14] Dohlie K (2010) Exploring industry needs ‘environmental footprint’ in fifth place. DNV container ship update N°. 1 2010 p. 14 a 17

[CR15] Ge J, Zhu M, Sha M, Notteboom T, Shi W, Wang X (2019). Towards 25,000 TEU vessels? A comparative economic analysis of ultra-large containership sizes under different market and operational conditions.

[CR16] Gomez Garay G (2014). La simulación marítima y su incidencia en seguridad, efectividad, confiabilidad y ahorro de recursos económicos en la operación portuaria, FORCE technology Dinamarca, IX Congreso Argentino de Ingeniería Portuaria May 5–7, Buenos Aires, Argentina.

[CR17] Gomez Paz M (2013) Diseño y aplicación de una metodología prospectiva Para la determinación de los condicionantes futuros del crecimiento de los grandes buques portacontenedores. Tesis (Doctoral), E.T.S.I. Caminos, Canales y Puertos (UPM) http://oa.upm.es/20924/

[CR18] Gomez Paz M, Camarero A, Gonzalez N (2015). Use of the Delphi method to determine the constraints that affect the future size of large container ships. Marit Policy Manag.

[CR19] Haralambides HE (2019). gigantism in container shipping, ports, and global logistics: a time-lapse into the future. Marit Econ Logistics.

[CR20] Huang S-T, Yoshida S (2013) Analysis of key factors for formation of strategic alliances in liner shipping company: service quality perspective on Asia/Europe route after global economic crisis; international journal of social, Behavioural, educational, economic, business and industrial engineering, vol 7,p 6

[CR21] IMO (2018) Adoption of the initial IMO strategy on reduction of GHG emissions from ships and existing IMO activity related to reducing GHG emissions in the shipping sector. https://unfccc.int/sites/default/files/resource/250_IMO%20submission_Talanoa%20Dialogue_April%202018.pdf

[CR22] Lim S-M (1998). Economies of scale in container shipping; maritime policy and management.

[CR23] MPA Singapore (2020). The world single largest container port – Singapore’s resilient port of the future. IAPH 2020 Sustainability awards resilient infrastructure.

[CR24] Notteboom T, Rodrigue J-P, De Monie G, Hall PV, McCalla B, Comtois C, Slack B (2009). The organizational and geographical ramifications of the 2008-09 financial crisis on the maritime shipping and port industries; chapter in. Integrating seaports and trade corridors.

[CR25] Penfold A (2008). Trade concentration and large vessels in the container trades.

[CR26] Penfold A (2017) The politics of trade and pressures on the supply chain – economic, political, and environmental issues. WSP Parsons Brinckerhoff http://aapa.files.cms-plus.com/2017Seminars/17Spring/Andre%20Penfold%20Final.pdf

[CR27] Podesta F (2000) Recent developments in quantitative comparative methodology: the case of pooled time-series cross-section analysis. McDonough School of Business, Georgetown University

[CR28] Portal Portuario (2020). MSC plantea intención de Traer buques de 400 metros de eslora a Chile. February 27th.

[CR29] Russo D (2018). Raising the baseline for ULCV. Maritime impact our expertise in stories DNV-GL.

[CR30] Sánchez RJ, Barleta EP (2018). Reflexiones sobre el futuro de los puertos de contenedores por el nuevo comportamiento de la contenedorización; Boletín FAL N° 366.

[CR31] Sánchez RJ, Chauvet P (2020). Contratos de concesión: fallas, obstáculos y efectos sobre la competencia; ITI series no 150.

[CR32] Sánchez RJ, Perrotti DE (2012). Looking into the future: big full containerships and their arrival to south American ports. Marit Policy Manage.

[CR33] Sánchez RJ, Wilmsmeier G (2017). Port management implications from economies of scale in the liner container shipping industry; chapter in Stephen Pettit, Anthony Beresford: Port Management - Cases in Port Geography, Operations, and Policy.

[CR34] Stopford M (2009). Forecasting - an impossible job? Clarkson research.

[CR35] Stopford M (2019) Coming to terms with the new era for shipping and ports. Martin Stopford, ESPO conference, Livorno May 23rd, 2019. https://globalmaritimehub.com/wp-content/uploads/2019/06/23-05-1100-Martin-Stopford.pdf

[CR36] STX (2008) Development of the World’s first 22,000 TEU class container ship, STX news, dream & future 85. Korea

[CR37] SYS C (2009). Measuring the degree of concentration in the container liner shipping industry.

[CR38] Taneja P, Walker WE, Ligteringen H, Van Schuylenburg M, Van Der Plas R (2010). Implications of an uncertain future for port planning. Marit Policy Manag.

[CR39] UNCTAD (1985) Port development: a handbook for planners in developing countries, United Nation publication. United Nations Conference on Trade and Development, Geneva,

[CR40] UNCTAD (2009) Review of Maritime Transport Series. https://unctad.org/en/pages/PublicationWebflyer.aspx?publicationid=2563

[CR41] Van Dorsser C, Taneja P, Vellinga T (2018). Port Metatrends: Impact of long term trends on business activities, spatial use and maritime infrastructure requirements in the Port of Rotterdam.

[CR42] Wang H (2011). Decoupling measure between economic growth and energy consumption of China. Energy Procedia.

[CR43] Wei YY, Hui SL (2019). Next-generation mega container ports: implications of traffic composition on sea space demand. Marit Policy Manag.

[CR44] Wijnolst N, Scholstens M, Waals F (1999). MALACCA MAX-the ultimate container carrier.

[CR45] Wilmsmeier G, Sánchez RJ, Gonzalez Aregall M, Monios J, Wilmsmeier G (2017). Before the (maritime) “hangover”? Chapter in. Maritime Mobilities.

